# The impact of organisational characteristics of staff and facility on infectious disease outbreaks in care homes: a systematic review

**DOI:** 10.1186/s12913-022-07481-w

**Published:** 2022-03-15

**Authors:** A. E. M. Liljas, L. P. Morath, B. Burström, P. Schön, J. Agerholm

**Affiliations:** Department of Global Public Health, Karolinska Institutet, Solna, Sweden

**Keywords:** Care homes, Long-term care, Older adults, Aging, Infectious disease outbreaks, Staff characteristics, Facility characteristics, COVID-19

## Abstract

**Background:**

Infectious disease outbreaks are common in care homes, often with substantial impact on the rates of infection and mortality of the residents, who primarily are older people vulnerable to infections. There is growing evidence that organisational characteristics of staff and facility might play a role in infectious disease outbreaks however such evidence have not previously been systematically reviewed. Therefore, this systematic review aims to examine the impact of facility and staff characteristics on the risk of infectious disease outbreaks in care homes.

**Methods:**

Five databases (MEDLINE, EMBASE, ProQuest, Web of Science, CINAHL) were searched. Studies considered for inclusion were of any design reporting on an outbreak of any infectious disease in one or more care homes providing care for primarily older people with original data on: facility size, facility location (urban/rural), facility design, use of temporary hired staff, staff compartmentalizing, residence of staff, and/or nursing aides hours per resident. Retrieved studies were screened, assessed for quality using CASP, and analysed employing a narrative synthesis.

**Results:**

Sixteen studies (8 cohort studies, 6 cross-sectional studies, 2 case-control) were included from the search which generated 10,424 unique records. COVID-19 was the most commonly reported cause of outbreak (*n* = 11). The other studies focused on influenza, respiratory and gastrointestinal outbreaks. Most studies reported on the impact of facility size (n = 11) followed by facility design (*n* = 4), use of temporary hired staff (*n* = 3), facility location (*n* = 2), staff compartmentalizing (n = 2), nurse aides hours (n = 2) and residence of staff (n = 1). Findings suggest that urban location and larger facility size may be associated with greater risks of an infectious disease outbreak. Additionally, the risk of a larger outbreak seems lower in larger facilities. Whilst staff compartmentalizing may be associated with lower risk of an outbreak, staff residing in highly infected areas may be associated with greater risk of outbreak. The influence of facility design, use of temporary staff, and nurse aides hours remains unclear.

**Conclusions:**

This systematic review suggests that larger facilities have greater risks of infectious disease outbreaks, yet the risk of a larger outbreak seems lower in larger facilities. Due to lack of robust findings the impact of facility and staff characteristics on infectious disease outbreaks remain largely unknown.

**Prospero:**

CRD42020213585.

**Supplementary Information:**

The online version contains supplementary material available at 10.1186/s12913-022-07481-w.

## Introduction

Infectious disease outbreaks are common in shared living spaces such as care homes [1]. Infection outbreaks in care homes often have a substantial impact on the rates of infection and mortality of the residents who primarily are frail older people with chronic physical, mental and/or cognitive conditions and thus more vulnerable to infections [2, 3]. The most common types of infectious disease outbreaks in care homes are respiratory infections such as influenza viruses, and gastrointestinal infections (often caused by noroviruses) [4]. Additionally, in the last 1.5 years numerous care homes worldwide have been severely impacted by COVID-19 (SARS-CoV-2), an acute respiratory syndrome with worse outcomes for older adults with multimorbidity compared to younger adults [5]. In several countries such as the United States, England, France, Spain, and Sweden, care home residents have been reported to account for 30–50% of all COVID-19-related deaths [6–8].

The prerequisites for infection control vary between care homes (also known as nursing homes or residential long-term care facilities) and depend on a range of factors including organisational factors such as facility characteristics (i.e. the physical building) [9] and staff characteristics (e.g. staff compartmentalizing, temporary hired staff) [10]. In the last few decades there has been a shift from providing traditional nursing home wards with hospital-like features such as shared rooms and no private space towards building care homes where residents have a single room and a common living room in a homelike atmosphere with an interior familiar to them [11]. Providing a homelike environment is considered particularly important to residents with dementia as hospital-like environments can contribute to spatial disorientation, stress and anxiety [11]. The homelike environment has however been reported an obstacle in infection prevention and control [12]. Unlike hospitals, care homes with a homelike environment have no transaction windows into the residents’ apartments and are challenged by facility characteristics such as rarely having a designated space where employees can remove personal protective equipment or get changed after having had close personal contact with an infected resident. Besides providing a homelike atmosphere, care homes are often designed to bring people together for social activities and meals, routines that might have to change during an infectious disease outbreak, with consequences such as loneliness and anxiety among the residents [13]. The design of care homes also affects the possibility of isolating residents or conducting cohort care to prevent transmission of infection during an outbreak. Handling residents with dementia or mental illness who have a strong compulsion to walk becomes particularly challenging when practising isolation and social distancing [14]. Earlier systematic reviews focusing on facility characteristics have shown that residents in care homes with a higher proportion of private rooms have reported better quality of life [15], and that care homes with fewer beds scored higher on service quality [16]. Findings on links between smaller facility size and residents’ quality of life were however inconsistent [15]. The latter systematic review further reported higher quality of life among the residents in care homes in rural areas [15]. However, no systematic review has investigated the impact of these and other facility characteristics such as the facility design on infectious disease outbreaks. Further, in a brief summary of evidence on how COVID-19spreads can be contained in care homes, the authors concluded that movement of staff between care homes should be limited, and that temporary staff are a key potential source of infection [17]. Similarly, a systematic review has reported higher risks of infection in healthcare settings with fewer registered nurses and more nursing aide staff, and with a higher proportion of temporary staff [18]. In the wake of the COVID-19 pandemic, the possible effects of care home staff, particularly nurse aides, working in multiple facilities on infection control has been debated [19]. Staff characteristics in relation to outbreaks of infectious diseases in care homes have however not previously been systematically reviewed. The impact of staff and facility characteristics are less studied aspects in healthcare research [20], yet considered potential modifiable factors for infection control in care homes [9, 10]. This includes community-acquired infectious diseases such as COVID-19 where the spread in the local community might result in infected care workers being the probable source of outbreaks in care homes [21]. Identifying factors of particular importance for infection control in the home care setting is essential to mitigate the spread of infectious diseases. Therefore, we have conducted a systematic review that aims to examine the impact of organisational features such as staff characteristics and facility characteristics on the risk of infectious disease outbreaks in care homes for older adults.

## Methods

### Search strategy

Five electronic databases were searched for this review: MEDLINE (Ovid), EMBASE, Sociological Abstract (ProQuest), Web of Science Core Collection and CINAHL (Ebsco). The systematic search was performed in April 2021. Key search terms were developed in collaboration with librarians and included long term care, infection, and disease outbreaks, and are presented in Appendix 1. The research protocol has been registered with PROSPERO (identification number CRD42020213585).

### Eligibility criteria

In this review, the outcome was an infectious disease outbreak in a care home, referring to one or more residents being infected. A single case of an infectious disease can be considered an outbreak if the disease is rare or has serious public health implications [22]. Table 1 presents the PICO (Population, Intervention, Comparison, Outcome) inclusion and exclusion criteria for eligible studies. Studies included were of quantitative, qualitative and mixed-method design reporting on an infectious disease outbreak in one or more care homes providing care for primarily older adults (≥65 years) with original data on one or more of the exposure features: facility size (typically measured as number of beds), facility location (urban/rural), the design or room layout of the facility, temporary hired staff, staff compartmentalizing (i.e. dividing staff at the care home facility into zones or units to prevent the spread of infection), residence of staff (home address of staff), and nursing aides hours per resident (number of hours certified nurse aides are available to care for residents and undertake administrative work). The seven exposure features assessed were chosen based on facility and staff characteristics identified by the authors prior to the database search, and not previously systematically reviewed. The search was not restricted to certain years or languages. Reviews and commentaries with no original data were excluded.

**Table 1:** PICO

Population (Setting)	Care homes for primarily older adults (aged ≥65 years) defined as a facility with 24 h surveillance and access to some level of medical care within the facility.
Intervention (Exposure)	Facility size, facility location, facility design, use of temporary hired employment, staff compartmentalizing, residence of staff, and nurse aides hours per resident.
Comparison	No restriction
Outcome	An infectious disease outbreak (at least 1 resident infected in the care home facility). Studies that included disease outbreaks of non-infectious or non-communicable diseases were excluded.

### Study selection, data extraction and quality assessment

References retrieved were downloaded to EndNote X9 reference management and Rayyan for systematic reviews and independently screened for eligibility by two researchers (LM and AL). Any disagreements were resolved through discussion between the reviewers and with a third researcher (JA). Researchers LM and AL extracted the data together and organised the studies according to study outcome using a standardised data extraction form [23]. The two researchers then assessed the studies for quality using the critical appraisal skills Programme (CASP) (https://casp-uk.net/casp-tools-checklists/). The appraisal tool is used to analytically evaluate whether the results are valid, biases have been minimised and confounding factors have been considered, with the answer options yes/no/can’t tell. Studies that were given one or more ‘no’ or ‘can’t tell’ answers in the quality assessment were read again and discussed with the third researcher. If any ‘no’ or ‘can’t tell’ answers remained, the study was considered not to be of high quality and subsequently excluded (Appendix 2). A narrative synthesis allowed for the findings of the heterogenous studies included in the review to be compiled.

## Results

### Results of the systematic search

**Fig. 1:**
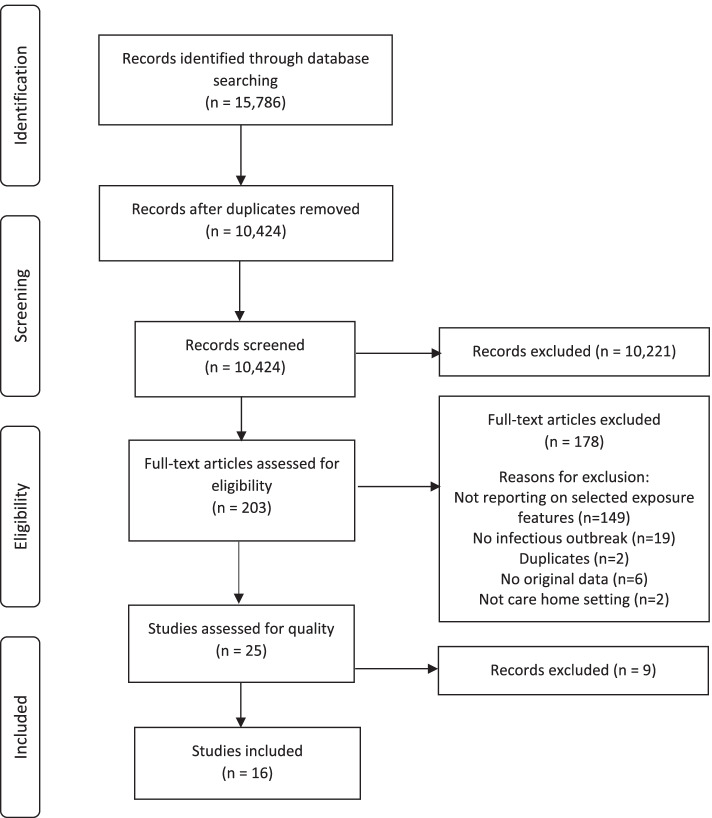
PRISMA flow chart

A PRISMA flow chart of the screening process is presented in Fig. 1. The search yielded 15,786 records and after removal of duplicates 10,424 records remained, which were screened for eligibility. Two hundred and three studies were read in full text of which 178 studies were excluded, mainly because they did not report on any of the exposure features of interest. The remaining 25 studies were assessed for quality using the CASP tool after which 9 studies were excluded, resulting in a total of 16 studies included in the review. Results on each exposure feature in each individual study were summarised and presented in tables (Tables 2 and 3).

**Table 2:** Findings on facility size

No association between facility size and infectious disease outbreak	Facility size and risk of an outbreak	Facility size and larger outbreaks	Facility size and the extent of the outbreak
Bowblis (2020) (N = 292) Number of beds consistently showed no association with the likelihood of having at least one resident infected in COVID-19.	He (2020) (*N* = 1223) Larger homes with higher bed occupancy were positively associated with having one or more COVID-19 case (OR: 1.009, 95%CI 1.006–1.012) and COVID-19 mortality (OR 1.006, 95%CI 1.003–1.009). Adjusted for ownership and years of operation.	Bowblis (2020) (*N* = 292) Facilities with a larger number of beds were less likely to report a high number of cases at two of three time points (marginal effects for April: mean − 0.012, SE 0.010; May: mean − 0.025, SE −0.012 (*p* < 0.05); June; mean − 0.032, SE 0.007 (*p* < 0.01)). Adjusted for facility structural, occupancy and payer-mix, resident and case-mix characteristics, and rurality.	Inns (2018) (*N* = 379) The size of the facility was associated with the duration of the outbreak with larger care homes having longer lasting outbreaks (IRR 1.426, 95%CI 1.275–1.595, *p* < 0.001). Adjusted for total outbreaks, winter outbreaks, care home quality rating, bed-to-staff ratio, residents with dementia and closure of home within 3 days.
Halloran (2020) (*N* = 154) No difference in the risks of an influenza outbreak between smaller homes (< 30 residents) and larger homes (> 30 residents) (*p* = 0.65).	Li (1996) (*N* = 171) A greater number of beds was associated with nosocomial respiratory and gastrointestinal disease outbreaks (RR 1.005, 95%CI 1.002–1.009). Adjusted for infection control actions such as medical protocols and laboratory results, and authorities’ area offices.	Halloran (2020) (*N* = 154) Compared to smaller facilities, larger facilities (≥51 residents) had a lower risk of having residents with influenza like illness once an outbreak had been declared (RR 0.55, 95%CI 0.38–0.80, *p* < 0.001). Adjusted for dementia care, care home quality score and antiviral prophylaxis activation.	
	Lin (2011) (*N* = 748) Higher rates of norovirus outbreaks observed in larger care homes (RR 1.4, 95%CI 1.3–1.5, *p* < 0.0001). Adjusted for staff-to-resident ratio, age of residents, bedridden residents, wheelchair accessibility and partition between beds.	Shallcross (2021). (*N* = 5126) No difference observed when comparing smaller care homes (< 25 beds) with larger care homes on the likelihood of a large outbreak (defined as 1/3 residents infected) (25–50 beds OR 0.70, 95%CI 0.41–1.19; > 50 beds OR 1.13, 95%CI 0.66–1.96). Adjusted for social deprivation, provider type, staff-to-bed ratio, region, quality rating, staff sick pay, cohorting of staff, cleaning frequency, use of personal protective equipment, inability to isolate residents, new admissions and closure to visitors.	
	Lomardo (2020) (*N* = 1356) COVID-19 outbreak associated with facility size larger than the median of 60 beds (OR 1.50, 95%CI 1.09–2.07, *p* = 0.013). Adjusted for lack of personal protective equipment, lack of personnel, lack of information, difficulty transferring, difficulty isolating, lack of medication, beds-to-staff ratio and geographical area.	Stall (2020) (*N* = 623) Larger homes with more residents were protectively associated with the number of residents infected with COVID-19 (RR 0.84, 95%CI 0.73–0.95) and resident deaths in COVID-19 (RR 0.81, 95%CI 0.70–0.95). Adjusted for chain ownership and staff-to-bed ratio.	
	Morciano (2021) (*N* = 4428) Larger homes had higher risk of COVID-19 deaths per bed: small homes (0–23 beds) OR 2.2, 95%CI 1.8–2.7; medium homes (24–40 beds) OR 4.7, 95%CI 4.0–5.5; large homes (41+ beds) OR 8.6, 95%CI 7.3–10.0. Adjusted for dementia care, legal status and provider type.		
	Shallcross (2021) (*N* = 5126) Larger care homes (> 50 beds) were significantly more likely to have a COVID-19 outbreak compared to smaller care homes defined as < 25 beds (reference). Care homes with 25–50 beds OR 1.73, 95%CI 1.30–2.31; > 50 beds OR 2.76, 95%CI 1.97–3.88). Adjusted for social deprivation, provider type, staff-to-bed ratio, region, quality rating, staff sick pay, cohorting of staff, cleaning frequency, use of personal protective equipment, inability to isolate residents, new admissions and closure to visitors.		
	Stall (2020) (N = 623) Homes with larger numbers of residents were significantly associated with greater odds of an outbreak (OR 1.38, 95%CI 1.18–1.61). Adjusted for chain ownership and staff-to-bed ratio.		
	White (2020) (*N* = 3357) Larger facility (presented as a 10-bed difference in facility size) was associated with greater probability of having at least one resident with COVID-19 infection. Marginal effect: 0.90, SE 0.159, *p* < 0.001. Findings adjusted for county COVID-19 prevalence, date of first county case, and universal testing at facility.

**Table 3:** Findings on facility location, facility design, staff compartmentalizing, temporary hired staff, nurse aides hours, and residence of staff

Author, year	Any clarifications related to the outcome(s), and factors adjusted for statistically significant associations	Facility location	Facility design	Staff compartmentalizing	Temporary hired staff	Nurse aides hours per resident per day	Residence of staff
Bowblis, J. & Applebaum, R. (2020)	Measures assessed at three time points (April, May, June 2020) which were reported separately and combined. Findings adjusted for facility structural, occupancy and payer-mix, resident and case-mix characteristics, and rurality.	Care homes in rural areas, including rural cities, were consistently less likely to have a COVID-19 infected resident (marginal effect mean (SE) -0.117 (0.057), *p* < 0.05). There was no statistically significant association at any of the time points between rural location and having a high number of COVID-19 cases (defined as number of cases equal to at least 20% of beds) among facilities with at least one case.	–	–	No consistent effects over three months were observed for use of temporary hired staff on the likelihood of having a resident with COVID-19 infection or having a high number of COVID-19 cases.	No consistent effects over three months on certified nurse aides hours per resident day on the likelihood of having a resident with COVID-19 infection or having a high number of COVID-19 cases.	–
Drinka P.J. et al. (2004)	One building with more space per resident and 100% filtered air compared to three (older) buildings with fewer square feet per resident and 30–70% air circulated back into the buildings.	–	No significant differences in infectious outbreaks of Influenza A observed between the facility buildings in five subsequent years	–	–	–	–
Gorges, R.J. & Konetzka R.T. (2020)	High nursing aides hours defined as greater than 66th percentile of case-mix adjusted hours. Adjusted for facility size, ownership type, chain status, percentage of Medicaid residents, percentage of White residents, metropolitan status, and county cases per capita.	–	–	–	–	High nurse aides hours were not associated with a COVID-19 outbreak but associated with lower risks for a larger outbreak (OR 0.790, SE 0.058, *p* < 0.01)	–
He, M et al. (2020)	Facility age measured by years of operation. Adjusted for facility size and ownership type.	–	No significant associations between facility age and one or more cases of COVID-19.	–	–	–	–
Li, J. et al. (1996)	Multiple units refer to units in the same care home. Findings adjusted for infection control actions such as medical protocols and laboratory results, and authorities’ area offices.	–	–	Having staff working at multiple units increased the risk of a nosocomial respiratory or gastrointestinal disease outbreak (RR 2.51, 95%CI 1.07–5.89) compared to having multiple units with separate staff.	–	–	–
Lin, H. et al. (2011)	Number of outbreaks in care homes that supply isolation areas for infected residents part of infection control practices compared to care homes with no isolation areas	–	Having an isolation area was not associated with lower risk of norovirus outbreaks.	–	–	–	–
Rolland, Y. et al. (2020)	Staff compartmentalization defined as organization of the work so that the team works in small groups in one area of the care home with no physical connection with the other members of the team. Type of employment defined as permanent versus use of professional interim. Findings adjusted for care home administrative status and organization of the meals.	–	–	Staff compartmentalization was associated with lower risk of COVID-19 outbreak (OR 0.17 95%CI 0.04–0.67, p < 0.01).	Use of professional interim was not associated with COVID-19 infection.	–	–
Shallcross, L. et al. (2021)	Employment of other bank or agency staff used for nursing aides. Findings adjusted for social deprivation, provider type, staff-to-bed ratio, region, quality rating, staff sick pay, cohorting of staff, cleaning frequency, use of personal protective equipment, inability to isolate residents, new admissions and closure to visitors.	–	–	–	Temporary employment a few times per month (OR 1.28, 95%CI 1.20–1.37, p < 0.0001), a few times per week (OR 1.08, 95%CI 1.01–1.16, *p* = 0.022) and on most days or every day (OR 1.08, 95%CI 1.00–1.16, *p* = 0.044) were all associated with higher proportion COVID-19 infection among residents compared to having no temporary staff.	–	–
Shi, S. et al. (2020)	Home zip codes for all staff were obtained to assess the proportion of staff living in areas with high rates of COVID-19. Findings adjusted for resident characteristics (age, sex, medical conditions, activities of daily living scores, bowel incontinence, physical behaviours, and wandering.	–	–	–	–	–	Staff living in a community with a high rate of COVID-19 was a significant predictor of COVID-19 infections in the care homes (OR 1.06, 95%CI 1.04–1.08)
Stall, N. et al. (2020)	Older design refers to below year 1972 design standards of larger room size, private washroom and single-occupancy. Findings adjusted for number of residents, chain ownership and staff-to-bed ratio.	–	Older design standard (4-person rooms) was associated with greater risk of COVID-19 outbreaks among residents (RR 1.88, 95%CI 1.27–2.79)	–	–	–	–
Sugg, M. et al. (2020)	Population density was used as a proxy for urban location. Findings adjusted for ownership, quality rating, population employment rates, ethnic groups, household size, and income per capita.	A higher risk of COVID-19 outbreaks was observed in care homes located in areas with higher population density per square mile (rate ratio 1.10, 95%CI 1.00–1.20, *p* = 0.042) across the USA. When examining the results regionally, the association remained significant in only 13 states.	–	–	–	–	–

### Description of the included studies

The 16 studies included in this review are presented in Table 4. Eight studies were cohort studies of which 5 studies had obtained data during one single outbreak and 3 studies had collected data during two to six years. Six studies were of cross-sectional design and two studies were case-control studies. None of the included studies were of qualitative or mixed-methods design. The majority of studies (*n* = 11) covered COVID-19 outbreaks, two studies were on influenza outbreaks, two on gastrointestinal outbreaks, and one study on both respiratory and gastrointestinal outbreaks. Ten studies were undertaken in North America, one in Hong Kong and five in Europe.

**Table 4:** Characteristics of studies included

Author, year	Country (State/Region)	Study design and study period	Data source	Study setting	Number of care homes	Infectious disease	Outcome of interest
Bowblis, J. & Applebaum, R. (2020) [24]	United States of America (USA), (Ohio)	Cohort study, data collected April–June 2020	Ohio Department of Health, Nursing Home Compare database, and Payroll-based Journaling	Nursing homes that have reported at least one case of COVID-19 to Ohio Department of Health.	942	COVID-19	Facility size, facility location, temporary hired staff, nurse aides hours
Drinka P.J. et al. (2004) [25]	USA (Wisconsin)	Cohort study, data collected during six influenza season 1993–2000	Objectilvey measured data collected by researchers	A four-building, long-term care facility for veterans and their spouses	1	Influenza A	Facility design
Gorges, R.J. & Konetzka R.T. (2020) [26]	USA	Cohort study, data obtained in June 2020	Nursing Home Compare (NHC) database, Long-Term Care Focus, and COVID-19 Nursing Home Dataset from the Centers for Medicare & Medicaid Services (CMS)	All nursing homes in the CMS COVID-19 Nursing Home Dataset that had passed the CMS Quality Assurance Check as of June 25, 2020.	13,167	COVID-19	Nurse aides hours
Halloran, N.F. et al. (2020) [27]	England (Cheshire and Merseyside)	Case-control study comparing characteristics between care homes with and without a declared influenza outbreak	Public Health of England Health Protection Case Management System, and the Care Quality Commission (CQC)	All CQC-registered care homes in Cheshire and Merseyside that declared an influenza outbreak between mid-December 2017 and May 2018	154	Influenza A and B	Facility size
He, M et al. (2020) [28]	USA (California)	Cross-sectional study, data obtained in June 2020	California Department of Public Health, Nursing Home Compare Database, and nursing home data from Long-Term Care Focus	All nursing homes in California	1223	COVID-19	Facility size, facility design
Inns, T. et al. (2018) [29]	England (Cheshire and Merseyside)	Cohort study, data collected December 2012–December 2016	Records collected by the local Community Infection Prevention & Control Team, and data from the Care Quality Commission (CQC)	All CQC-registered care homes in Cheshire and Merseyside	379	Infectious gastroenteritis	Facility size
Li, J. et al. (1996) [30]	USA (New York State)	Case-control study (case = care home with outbreak), all of year 1992	New York State Department of Health, and survey data completed by care home managers	All licensed nursing homes in New York State caring primarily for older people	171	Respiratory and gastrointestinal infection	Facility size, staff compartmentalizing
Lin, H. et al. (2011) [31]	Hong Kong	Cohort study from January 2005 until December 2007	Annual Territorywide Infection Control Checklist Survey, and the Public Health Information System	All elderly homes operating in Hong Kong	748	Norovirus	Facility design
Lombardo F.L. et al. (2020) [32]	Italy	Cross-sectional study, survey completed March–May 2020	Survey data provided by care home directors	All nursing homes in Italy	1356	COVID-19	Facility size
Morciano, M. et al. (2021) [33]	England	Cohort study, data obtained in August 2020	Data records from the Care Quality Commission	All nursing homes in England with death notification data	4428	COVID-19	Facility size
Rolland, Y. et al. (2020) [34]	France (Haute-Garonne)	Cross-sectional study, survey completed in March–May 2020	Survey data provided by care home directors	All long-term care facilities in Haute-Garonne	124	COVID-19	Temporary hired staff, staff compartmentalizing
Shallcross, L. et al. (2021) [35]	England	Cross-sectional study, telephone-survey completed in May–June 2020	Survey data provided by care home directors, SARS-CoV-2 RT-PCR test results	All long-term care facilities for individuals aged ≥65 years or providing dementia care	5126	COVID-19	Facility size, temporary hired staff
Shi, S. et al. (2020) [36]	USA (Boston, Massachusetts)	Retrospective cohort study, data collected March–May 2020	Medical records, SARS-CoV-2 test results, federally mandated clinical assessment data of residents	An approximately 500-bed academic long-term care facility in Boston	1	COVID-19	Residency of staff
Stall, N. et al. (2020) [37]	Canada (Ontario)	Retrospective cohort study, data collected March–May 2020	Ontario Ministries of Health and Long-Term Care, COVID-19 Ontario Census	All long-term care homes in Ontario	623	COVID-19	Facility size, facility design
Sugg, M. et al. (2020) [38]	USA	Cross-sectional study of a national cohort, data obtained in June 2020	Centers for Medicare & Medicaid Services, American Community Survey, the 2010 Census, SARS-CoV-2 test results	All nursing homes operating since 2015	13,709	COVID-19	Facility location
White, E.M. et al. (2020) [39]	USA	Cross-sectional study, data obtained in May 2020	Genesis Healthcare database, Long-Term Care Focus, Nursing Home Compare database	Skilled nursing facilities by the provider Genesis HealthCare and other providers operating in states providing data on COVID-19 test results	3357	COVID-19	Facility size

#### Facility size

Eleven studies reported on facility size in relation to risk of infection outbreak of which several reported on multiple aspects of facility size as shown in Table 2. Definition of ‘large’ facility varied between the studies and is presented in Table 2 for each study, where available. Two studies reported no association between facility size and risk of infectious disease outbreak [24, 27]. Most studies (8 of 11) reported an association between larger facility size and greater risk of an outbreak [28, 30–33, 35, 37, 39]. Four studies also reported on larger outbreaks in relation to facility size [24, 27, 35, 37] of which all but one showed a lower risk of a large outbreak in larger care homes [35]. In one study the size of the care home was further associated with the duration of the outbreak with larger care homes having longer lasting outbreaks [29].

#### Facility location

Two studies assessed the risk of a COVID-19 outbreak in relation to the care homes being in an urban or rural area [24, 38]. Both studies showed that care homes located in urban areas were more likely to report cases of COVID-19 of which one of the studies provided findings for 13,709 care homes across the USA [38]. In their study, they used Census 2010 data to calculate population density per square mile and used population density as a proxy for rurality. When examining the results regionally, the association remained significant in 13 states [38]. The other study was conducted in Ohio [24], one of the states in which the findings by Sugg et al. (2020) [38] remained when examined regionally. In the study from Ohio by Bowblis & Applebaum (2020) [24], measures were taken once a month for three months (April–June 2019) and consistently showed that care homes in rural areas, including rural cities, were less likely to have a COVID-19 outbreak and less likely to have a larger outbreak (defined as at least 20% of residents infected) among facilities with at least one case.

#### Facility design

The influence on the facility design of the care homes on infectious disease outbreaks was examined in four studies focusing on various design aspects [25, 28, 31, 37]. A study from the Canadian province Ontario showed that multi-occupancy rooms, more common in older than newer care homes, were associated with an almost two-fold risk of a COVID-19 outbreak compared to single rooms (RR 1.88, 95%CI 1.27–2.79) [37]. In a study conducted in California, U.S., facility age measured as years of operation was not associated with an outbreak of one or more cases of COVID-19 (OR 1.006, 95%CI 0.995–1.017) [28]. Similarly, another North American study also comparing older and newer buildings where the new building had installed filters preventing recirculation of air, showed no significant differences in influenza outbreaks observed between the buildings in five subsequent years [25]. Further, having isolation areas for infected residents to control infection was not associated with lower risk of norovirus outbreaks in a study of 748 care homes in Hong Kong [31].

#### Staff compartmentalizing

Two studies reported on staff compartmentalizing i.e. dividing staff at the care home facility into zones or units to prevent the spread of infection [30, 34]. One of the studies reported 2.5 times higher risks of nosocomial respiratory or gastrointestinal disease outbreaks if staff worked at multiple units compared to having staff compartmentalized (RR 2.51, 95%CI 1.07–5.89) [30]. The other study was conducted during the COVID-19 pandemic and showed that arranging for staff compartmentalizing within zones resulted in a significantly lower likelihood of having any confirmed COVID-19 cases (OR 0.17, 95%CI 0.04–0.67) [34].

#### Temporary hired staff

Three studies reported on use of temporary hired staff and the likelihood of COVID-19 outbreaks in care homes [24, 34, 35]. The largest of these three studies (care homes *n* = 5126) showed that use of temporary staff was associated with higher proportion COVID-19 infection among residents compared to having no temporary staff, with the strongest association in care homes that only had temporary staff a few times per month (OR 1.28, 95%CI 1.20–1.37, *p* < 0.0001) [35]. The other two studies reported no association between use of temporary staff and COVID-19 outbreaks.

#### Nurse aides hours

The influence of nurse aides hours per resident on disease outbreaks was investigated in two studies of which both reported on COVID-19 outbreaks [24, 26]. Whilst one of the studies showed no consistent effects over three months of nurse aides hours and the number of COVID-19 cases [24], the other study reported no association between high nurse aides hours and an outbreak; yet showed an association between high nurse aides hours and lower risks for a larger COVID-19 outbreak [26].

#### Residence of staff

Only one study examined the influence of the residence of staff on infectious outbreaks in care homes. The study was conducted at a large care home (approximately 500 beds) and showed that staff living in a community with a high rate of COVID-19 was a significant predictor of COVID-19 outbreaks [36].

## Discussion

In this review, a range of organisational facility and staff characteristics assessed in earlier studies on infectious disease outbreaks in care homes have been reported. The findings suggest that urban location and larger facility size may be associated with greater risks of an infectious outbreak. The findings also suggest that the risk of a larger outbreak may be lower in larger facilities. Whilst staff compartmentalizing may be associated with lower risk of outbreak, staff residing in highly infected areas may be associated with greater risk of an outbreak. The influence of facility design, use of temporary staff, and nurse aides hours on infectious outbreaks remains unclear.

There is growing evidence that urban location and larger facilities increase the risk of infectious disease outbreaks in care homes [40, 41]. Larger care home facilities imply managing a greater number of residents including person-to-person contact with a larger number of different residents, staff and visitors, creating opportunities for infectious outbreaks [30]. The greater risk of infectious outbreaks that larger care homes seem to face may further reflect the community spread of infection [41]. As shown in this review, there is some evidence that staff residing in highly infected areas increases the risk of an outbreak at the care home where they work [36]. Previous research has also shown a link between the risk of an outbreak at a care home and the incidence of COVID-19 in the surrounding region [37]. Several earlier studies have implied that staff might contribute to the spread of infection [37, 41, 42]. Beside staff-to-resident and staff-to-staff transmission at the same care home, many care workers often hold second jobs and provide care to family members, which may increase the risk of transmission [42]. They also tend to feel obligated to come to work even when ill, due to low incomes and limited benefits [42]. It has been speculated that spread of infection due to staff working at multiple facilities is most likely to occur among facilities using temporary staff who provide services at multiple care homes [24]. However, findings from the three included studies on use of temporary staff are inconsistent with only one study reporting such an association [35]. This might be explained by the unclarity in whether the outbreaks studied were caused by community transmissions or pre-existing asymptomatic cases within the facility.

Apart from suggesting that larger facility size may be associated with greater risks of an infectious outbreak, this review also suggests that the risk of a larger outbreak seems lower in larger facilities. This might be explained by larger care homes often being purpose built allowing for staff compartmentalizing, suggesting that larger facilities have less crossover of staff between residents [27, 37]. Larger facilities might also have the advantage of ‘economy of scale’ in terms of greater resources (e.g. personnel and financial resources) allowing for high nurse aides hours. According to the findings of this systematic review, high nurse aides hours might not prevent outbreaks yet seem to reduce the risks of a larger outbreak. This supports a recent study showing that care homes that met the minimum staffing standards are likely to be able to prevent or delay COVID-19 resident infections [10]. Having enough nurse aides is considered crucial as implementing infection control such as laboratory testing and staff compartmentalizing at care homes is difficult without sufficient staffing levels [26]. This denotes strong and close links between facility and staff characteristics implying the need to apply a holistic perspective when studying disease outbreaks at care homes.

The included studies on facility design assessed different aspects: multi-occupancy rooms, older facility design, air circulation and isolation areas, aspects which all have been considered important in recent analyses of COVID-19 and the care home environment [9, 43]. Yet, the included studies reported no association on these aspects except multi-occupancy rooms, making it difficult to draw any conclusions on the influence of facility design on infectious outbreaks. Further well-designed studies are needed on the influence of the many aspects of facility design on infectious outbreaks to provide an understanding of their potential impact. Further research is also needed on staff and facility characteristics of large facilities in relation to infectious disease outbreaks, and should include tracking cases to provide possible explanations to this review’s suggested findings on facility size.

### Strengths and limitations

A major strength of the study is the novelty of systematically reviewing multiple organisational facility and staff characteristics on infectious disease outbreaks in care homes, a setting that has become increasingly important following the COVID-19 pandemic. The search strategy comprised of seven different outcome features of both facility and staff characteristics, was not restricted to a certain language or time period, and was applied to five different databases. Limitations include that the search did not include grey literature, possibly resulting in relevant data not being considered. Initially we intended to include care homes for older adults only however most studies did not specify the age of the residents making such restriction impossible. Further, whilst the search was not restricted to a certain infectious disease, most of the included studies focus on COVID-19, possibly negatively affecting the generalisability of the findings as COVID-19 has higher severe disease and mortality rates than e.g. influenza. However, not restricting the review to a certain infection is not necessarily a weakness per se. For instance, the lack of associations between facility design and infectious outbreaks in studies not focusing on COVID-19 might indicate that at least some aspects of facility design could be of greater importance to certain specific infections. Furthermore, the included studies did not report whether the care homes targeted provided a hospital-like or homelike environment. It is possible that the hospital-like model positively influences the risk of an infectious disease outbreak, making it difficult to conclude whether the findings are more applicable to a certain environment. Additionally, the methodological heterogeneity of the studies and the fact that the number of eligible studies was small makes it impossible to draw any firm conclusions. However, as the features addressed in the included studies may have an impact on infection control and furthermore are amenable to interventions, further studies are strongly warranted.

## Conclusions

In conclusion, this systematic review suggests that larger facilities have greater risks of infectious outbreaks, yet the risk of a larger outbreak seems lower in larger facilities. Following the COVID-19 pandemic, care home managers worldwide are preparing for new infectious outbreaks. This research could help inform policy of future care homes and care home managers in their preparation of future infectious outbreaks. However, evidence showing what actions on organisational characteristics are effective, is needed. Due to lack of robust findings the impact of facility and staff characteristics on infectious outbreaks remain largely unknown. Further research is needed to establish the effect of organisational characteristics of staff and facility on infectious disease outbreaks in care homes.

## Supplementary Information

**Additional file 1: Appendix 1.** Search terms used

**Additional file 2: Appendix 2.** Results from the CASP quality assessment of the non-included studies presented question by question.

## Data Availability

All relevant data are included in the article and/or its supplementary information files.
