# Correction to: The impact of organisational characteristics of staff and facility on infectious disease outbreaks in care homes: a systematic review

**DOI:** 10.1186/s12913-022-07796-8

**Published:** 2022-03-23

**Authors:** A. E. M. Liljas, L. P. Morath, B. Burström, P. Schön, J. Agerholm

**Affiliations:** Department of Global Public Health, Karolinska Institutet, Solna, Sweden

**Correction to: BMC Health Serv Res 22, 339 (2022)**

**https://doi.org/10.1186/s12913-022-07481-w**

Following publication of the original article [1], the authors identified an error in the author name of A. E. M. Liljas. The given names and family name were erroneously transposed.

The incorrect author name is: Liljas AEM

The correct author name is: A. E. M. Liljas

The author group has been updated above and the original article [1] has been corrected.

## References

[1] Liljas AEM (2022). The impact of organisational characteristics of staff and facility on infectious disease outbreaks in care homes: a systematic review. BMC Health Serv Res.

