# Supplementary material for: The impact of organisational characteristics of staff and facility on infectious disease outbreaks in care homes: a systematic review
Source: BMC Health Serv Res. 2022 Mar 15;22:339. doi: 10.1186/s12913-022-07481-w (PMC8921437; doi:10.1186/s12913-022-07481-w)
Supplement: Supplementary file 1 — Additional file 1: Appendix 1. Search terms used [file 12913_2022_7481_MOESM1_ESM.docx]

**Appendix 1. Search terms used**

Population

Aged

Aging

(aged or ageing or aging or centenarian* or elder* or geriatr* or nonagenarian* or octogenarian* or old age or old* adult* or old* individual* or old* men or old* man or old* patient* or old* people or old* person* or old* population* or old* woman or old* women or oldest old or septuagenarian*)

Care homes

Homes for the Aged

Long-Term Care

Residential Facilities

(long term care or nursing facilit* or nursing home* or residential facilit*)

Infectious disease

Cross Infection

Disease Outbreaks

Communicable Diseases

Disease Transmission, Infectious

Communicable Disease Control

((control* or cross or healthcare or health care or hospital or nosocomial) adj3 infection*)

((healthcare or health care or hospital or nosocomial) adj3 pneumonia*)

(disease* adj3 (communicable or infectious))

(epidemic* or outbreak* or pandemic* or transmission* or transmitted)

COVID-19

Coronavirus

Coronavirus Infections

(coronavirus* or corona virus* or covid* or ncov or sars-cov* or sarscov* or 2019ncov)
