# Supplementary material for: The impact of organisational characteristics of staff and facility on infectious disease outbreaks in care homes: a systematic review
Source: BMC Health Serv Res. 2022 Mar 15;22:339. doi: 10.1186/s12913-022-07481-w (PMC8921437; doi:10.1186/s12913-022-07481-w)
Supplement: Supplementary file 2 — Additional file 2: Appendix 2. Results from the CASP quality assessment of the non-included studies presented question by question. [file 12913_2022_7481_MOESM2_ESM.docx]

Appendix 2. Results from the CASP quality assessment of the non-included studies presented question by question.

| **Author (year)** | **Title** | **Did the study address a clearly focused issue?** | **Was the cohort recruited in an acceptable way?** | **Was the exposure accurately measured to minimise bias?** | **Was the outcome accurately measured to minimise bias?** | **Have the authors identified all important confounding factors?** | **Have they taken account of the confounding factors in the design and/or analysis?** | **Was the follow-up of subjects complete enough?** | **Was the follow-up of subjects long enough?** | **Do you believe the results?** | **Can the results be applied to the local population?** | **Do the results of this study fit with other available evidence?** | **What are the implications of this study for practice?** |
| --- | --- | --- | --- | --- | --- | --- | --- | --- | --- | --- | --- | --- | --- |
| Abrams et al. (2020) | Characteristics of U.S. Nursing Homes with COVID-19 Cases | Yes | Yes | Yes | Yes | No | No | No | No | Yes | Yes | Yes | Yes |
| Arden et al. (1995) | Vaccine use and the risk of outbreaks in a sample of nursing homes during an influenza epidemic | Yes | Yes | Yes | Yes | No | No | Yes | Yes | Yes | Yes | Yes | No |
| Bui et al. (2020) | Association Between CMS Quality Ratings and COVID-19 Outbreaks in Nursing Homes - West Virginia, March 17-June 11, 2020 | Can't tell | Yes | Yes | Yes | No | Yes | No | No | Yes | Yes | Yes | Yes |
| Chen et al. (2021) | Nursing Home Characteristics Associated With Resident COVID-19 Morbidity in Communities With High Infection Rates | Yes | Yes | Yes | Yes | Can't tell | No | No | No | Yes | Yes | Yes | Yes |
| Dean et al. (2020) | Mortality Rates From COVID-19 Are Lower In Unionized Nursing Homes | Yes | Yes | Yes | Yes | Yes | Yes | No | No | Yes | Yes | Yes | Can’t tell |
| Dutey-Magni et al. (2021) | COVID-19 infection and attributable mortality in UK care homes: Cohort study using active surveillance and electronic records (March-June 2020) | Yes | Yes | Yes | Yes | No | No | No | No | Yes | Yes | Yes | Yes |
| Trivedi et al. (2012) | Hospitalizations and mortality associated with norovirus outbreaks in nursing homes, 2009-2010 | Yes | Yes | Yes | Yes | No | No | No | No | Yes | Yes | Yes | Can't tell |
| Sun et al. (2020) | Predicting Coronavirus Disease 2019 Infection Risk and Related Risk Drivers in Nursing Homes: A Machine Learning Approach | Yes | Yes | Yes | Yes | No | No | No | No | Yes | Yes | Yes | Yes |
| Zimmerman et al. (2021) | Nontraditional Small House Nursing Homes Have Fewer COVID-19 Cases and Deaths | Yes | Yes | Yes | Yes | No | No | No | No | Yes | Yes | Yes | No |
